# Yoga as an Adjunct Treatment for Attention Deficit Hyperactivity Disorder (ADHD)

**DOI:** 10.1192/j.eurpsy.2023.1525

**Published:** 2023-07-19

**Authors:** S. Srinivas, S. Ashraf, A. Bachu, F. Kim, K. Shah

**Affiliations:** 1 A.J.Institute of Medical Sciences and Research Center, Mangaluru, Karnataka, India; 2 Northpointe Psychiatry, Lewisville, TX; 3 UAMS- Baptist Health, North Little Rock, AR; 4 Oklahoma State University/Griffin Memorial Hospital Psychiatry Residency Program, Norman, OK; 5 Wake Forest University, Winston-Salem, NC, United States

## Abstract

**Introduction:**

Attention Deficit Hyperactivity Disorder (ADHD) affects about 9.4% (6.4 million) children in the United States. Pharmacological treatment, including stimulants, is a known therapy for ADHD. However, its possible subtherapeutic effectiveness, residual symptoms, and adverse effects have prompted us to explore the current evidence of Yoga as an add-on therapy that has shown synergistic effects in recent studies.

**Objectives:**

1) We aim to assess Yoga’s efficacy as an add-on treatment for ADHD.

2) Assess the current evidence of Yoga as an add-on therapy for ADHD.

**Methods:**

We searched PubMed, PubMed Central, Medline, Web of Science, and Biosis databases with the keywords “Attention Deficit Disorder with Hyperactivity” (MeSH) and “ADHD” in the context of “Yoga” (MeSH). Identified 4 relevant studies that were published until September 30, 2022.

**Results:**

A study by Jensen et al. found improvement in impulsivity, hyperactivity, and restlessness in the ADHD with medications patient group after 20 Yoga sessions. Study utilized Conners’ Parents Rating Scales and found improvement in the Yoga group on Oppositional, Global Index Emotional Lability, Global Index Total, Global Index Restless/Impulsive, and ADHD Index scale, changes associated with the number of sessions (Jensen et al. J Atten Disord 2004; *7* 205-216). In another study, children with ADHD showed significant improvement after 8 weeks of 16 Yoga sessions in accuracy rate and reaction time in the Visual Pursuit Test and Determination Test (Chou et al. Peer J 2017; 5 e2883). A nine-year-old male case showed improvement in inattentive and hyperactive-impulsive symptoms on Vanderbilt Assessment Scales from parents and teachers after 6 months of Yoga practice (Gunaseelan et al. Cureus 2021; 5, e2883). Another 6-week Yoga intervention randomized trial in pre-school ADHD children showed significant improvement in hyperactivity and inattention with fewer distractibility errors of omission and faster reaction time (Cohen et al. J Dev Behav Pediatr 2018; 39 200).

**Conclusions:**

The results suggest that Yoga has beneficial effects as an adjunct treatment to pharmacotherapy in ADHD for reducing hyperactivity and inattentiveness. Additionally, studies indicate its effectiveness in managing stress, anxiety, energy levels, and impulse control by staying focused with the help of breath and mind control. To explore the full spectrum of benefits and effectiveness of Yoga as an add-on therapy for ADHD patients, we recommend robust well-design future studies.

**Disclosure of Interest:**

None Declared

